# Breaking the Therapeutic Ceiling: Dual Biologic or Small Molecule Therapy for Refractory Inflammatory Bowel Disease

**DOI:** 10.1093/crocol/otac005

**Published:** 2022-02-05

**Authors:** Waseem Ahmed

**Affiliations:** 1 University of Colorado Crohn’s and Colitis Center, Aurora, Colorado, USA; 2 Division of Gastroenterology and Hepatology, University of Colorado School of Medicine, Aurora, Colorado, USA

Over the past 3 decades, the advent of biologic and small molecule therapies has considerably improved the management of inflammatory bowel disease (IBD). Early use of these therapies in both moderate-to-severe Crohn’s disease (CD) and ­ulcerative colitis (UC) is associated with improved patient morbidity, with decreasing use of steroids and rates of surgery. Despite such advances, a significant proportion of patients remain nonresponders or have intolerances to current therapies.^1^ Furthermore, patients with extraintestinal manifestations, particularly those that do not correlate with intestinal disease activity, or concomitant immune-mediated disorders often have uncontrolled symptoms despite control of their luminal disease. While additional pathways involved in the pathogenesis of IBD and their potential therapeutic targets continue to be studied, even a brief review of registry clinical trials for all currently and soon-to-be approved therapies establishes a potential therapeutic ceiling, leaving patients with medically refractory disease with limited viable treatment options.^1^

This failure to achieve sustained remission with currently available treatments has led to an emerging strategy of combining mechanistically different biologics or small molecules for the treatment of medically refractory IBD. Targeting multiple pathways involved in disease pathogenesis may have an additive effect on treatment efficacy, while maintaining an acceptable safety profile. Numerous observational studies with limited patient sample sizes have recently demonstrated the successful use of this strategy; however, summary data to guide clinicians are lacking.^2–5^ In this issue of *Crohn’s & Colitis 360*, Alayo et al provide an updated systematic review and meta-analysis on the safety and efficacy of dual biologic therapy (DBT) or small molecule therapy combined with a biologic therapy (SBT) in IBD.

The authors identified 13 studies consisting of 266 patients who underwent 271 therapeutic trials of DBT or SBT, excluding single case reports and patients on drugs not currently FDA-approved for IBD. Additionally, corresponding authors of previously published data were contacted to provide raw data in instances of missing or incomplete data. The primary outcome of interest was safety assessed as pooled rates of adverse events (AEs) and serious AEs (SAEs) for each combination of therapy. Secondary outcomes of interest included pooled rates of clinical response and remission and endoscopic or radiographic response and remission. 71% of patients had CD and 28% UC, with the remaining having undifferentiated IBD. 55% of patients were female, and the median number of prior biologics across studies ranged from 0 to 4. The most common combinations of DBT and SBT were vedolizumab (VDZ) with a tumor necrosis factor (TNF) antagonist and VDZ with tofacitinib, providing each a total of 56 and 57 therapeutic trials.

Pooled rates of AEs in the above combinations were 24.1% (95% CI 11.8–38.4) for VDZ–TNF antagonist and 18.3% (95% CI 3.0–40.0) for VDZ–tofacitinib. Pooled rates of AEs for other combination therapies were similar. Pooled rates of SAEs for VDZ–TNF antagonist were 9.6% (95% CI 1.5–21.4) and 1.0% (95% CI 0.0–7.6) for VDZ–tofacitinib, with the majority of other combinations having a pooled rate of SAEs of 0%. Across all combinations, 75% (15/20) of SAEs were reported infectious complications. Pooled rates of clinical remission for therapeutic trials of VDZ–TNF antagonist were 55.5% (95% CI 19.6–88.5) and for VDZ–tofacitinib 47.8% (95% CI 19.0–77.4), with corresponding rates of endoscopic or radiographic remission of 18.0% (95% CI 1.6–41.8) and 24.6% (95% CI 6.4–47.6). Corresponding efficacy rates when analyses were limited to patients on dual therapy primarily due to active luminal disease were similar.

This updated analysis by Alayo et al offers granular data specific to individual combinations of dual therapy for refractory IBD. The authors are further commended for obtaining data not included in the initial publications of several included observational studies, allowing for the above pooled estimates on individual combination therapies. Similar to previous analyses, no new safety signals for any individual combination therapy were observed.^6,7^ Additionally, safety estimates for the various combinations are similar to reported estimates for biologics or small molecules when used as monotherapy. While prior RCTs on dual biologic therapy in rheumatologic disorders have previously demonstrated a higher frequency of AEs in patients on dual therapy, findings thus far in IBD are encouraging, likely owing to the favorable safety profiles of vedolizumab and ustekinumab.^8–10^ Granted, further longitudinal data are needed noting the inconsistency in the median follow-up period and high rate of discontinuation in the included studies. While the authors’ findings suggest potential efficacy for the use of dual therapies in medically refractory disease, significant limitations remain. The majority of included data are derived from observational studies prone to publication bias, and there was significant heterogeneity observed between studies with an overall very low certainty in evidence for all outcomes. The limited sample size of individual combinations further precluded any comparative analyses.

DBT or SBT remains a potentially effective option for patients with refractory IBD not candidates for established medical and surgical treatment options and demonstrates an acceptable safety profile. These patients are commonly excluded from clinical trials due to a number of prior therapies and other disease-related factors; furthermore, this population often has a history of repeated surgical resections and can be at risk for short bowel syndrome and parenteral nutrition dependence. However, RCTs designed to assess both safety and efficacy are required to confirm these data. The only RCT thus far on dual therapy investigated the safety and tolerability of combination infliximab and natalizumab, a strategy unlikely to be used in the future.^11^ Fortunately, additional prospective clinical trials using dual therapy are currently ongoing in both CD and UC.^12,13^

With currently available data, DBT or SBT should be individualized to only select patients with close monitoring in specialized IBD centers. Future areas of clinical interest include alternative forms of dual therapy, such as a combination of medical and nutritional/microbiome interventions, and the use of dual therapy in individuals with severe IBD naïve to medical therapy. While we continue to hope for individual therapies with improved rates of remission, no therapy to date has demonstrated consistently higher rates of clinical and endoscopic remission following the registration trials for TNF antagonists. Perhaps, the key to finally breaking the therapeutic ceiling lies in a combination approach.

## Data Availability

There are no primary data for this editorial.
